# Research on the Physico-Mechanical Properties of Moso Bamboo with Thermal Treatment in Tung Oil and Its Influencing Factors

**DOI:** 10.3390/ma12040599

**Published:** 2019-02-17

**Authors:** Tong Tang, Xiufang Chen, Bo Zhang, Xianmiao Liu, Benhua Fei

**Affiliations:** 1 Key Laboratory of Bamboo and Rattan Science and Technology of the State Forestry Administration, Department of Bio-materials, International Centre for Bamboo and Rattan, Futong Dong Dajie, Chaoyang District, Beijing 100102, China; tangtong@icbr.ac.cn (T.T.); liuxm206@163.com (X.L.); 2 Qingdao Institute of Bioenergy and Bioprocess Technology, Chinese Academy of Sciences, Songling road, Qingdao 266101, China; boews@sina.com

**Keywords:** tung oil, moso bamboo, oil heat treatment, mechanical properties

## Abstract

In this study, the effects of tung oil heat treatment on the physico-mechanical properties of moso bamboo were investigated. Here, heat treatment in tung oil at 100–200 °C was used to modify natural bamboo materials. The changes in the nanostructures of cell walls in bamboo caused by oil heat treatment, like density, chemical compositions, and cellulose crystalline, were evaluated to study their correlation with mechanical properties. Results showed that the mechanical performance of bamboo, such as ultimate stress, modulus of elasticity (MOE), and modulus of rupture (MOR), didn’t reduce after heat treatment below 200 °C, compared with the untreated bamboo, which was mainly due to the tung oil uptake, stable cellulose content, and the increment of cellulose crystalline. No remarkable change in the ultimate strain occurred for bamboo materials thermally treated below 140 °C, but it decreased obviously at the heating temperature over 180 °C, mainly due to the degradation of hemicellulose resulting in a decrease in the viscoelasticity of cell wall.

## 1. Introduction

Bamboo is one of the most abundant biomass resources, which has advantages, such as short growth cycle, lightweight, and good mechanical property [1,2]. As a renewable raw material, bamboo has been widely used in construction, building facade, decoration, and furniture [3,4,5,6,7]. However, bamboo has some inherent drawbacks, like hydrophilic property, dimensional instability, and low resistance to decay, which greatly shortens its service life [8,9]. Some efforts have been devoted to modifying bamboo to improve the less-beneficial features, including hydrophilic property, dimensional stability, and fungi resistance [10,11]. From the cost-effective, eco-friendly, and sustainable chemistry standpoint, oil heat treatment is considered to be one of the most effective approaches to ameliorate these drawbacks of bamboo materials, so it shows a great potential application in treating bamboo materials in the industry.

It remained the focus of controversy whether oil heat treatment would cause adverse effects on mechanical properties of wood or bamboo. Fang et al. studied canola oil heat treated densified aspen wood veneers at 180–220 °C for 1–3 h, and the result showed that bending modulus of elasticity (MOE) increased after oil heat treatment [12]. On the other hand, several studies found that oil heat treatment of wood and bamboo would result in a decrease in mechanical strength. For instance, Yang et al. reported that MOE and modulus of rupture (MOR) of moso bamboo would decrease after heat treatment in linseed oil over 150 °C for 2 h [13]. In industry, oil heat treatment of wood materials at a high temperature around 180–260 °C could improve the less-beneficial features of the natural lignocellulosic materials, but the cell structure and chemical components of wood would be altered permanently by oil heat treatment at a high temperature, probably resulting in the undesirable changes of mechanical properties [13,14]. In some studies, it had been demonstrated that the degradation of chemical components resulted in changes in the mechanical properties of wood [15,16]. Furthermore, the mechanical performance of oil heat treated bamboo might also be related to oil uptake. Cheng et al. reported that the oil uptake was propitious to MOR in static bending of bamboo [17]. However, the bamboo after oil heat treatment faced some knotty problems, such as moving out of oil and giving unpleasant smell during application. 

Tung oil, also known as China wood oil, has been extensively used to protect wood furniture and construction from fungi decay in China for over a thousand years [18]. Tung oil mainly contains unsaturated fatty acids of α-eleostearic acid (77–82%), oleic acid (3.5–12.7%), and linoleic acid (8–10%) [19]. The highly unsaturated, conjugated systems would make tung oil be oxidized by oxygen and rapidly polymerized to form an oily film in the inner walls or surface of bamboo [20], so tung oil would be stable in the bamboo during the use. Currently, few studies have systematically investigated the impact of tung oil heat treatment on the nanostructures of cell walls and mechanical properties of moso bamboo and their relationship. In comparison to the extensive studies about wood, the study of oil heat treatment on bamboo is relatively scarce. Although bamboo chemical compositions are similar to wood, the structure of bamboo has a comparatively heterogeneous structure with a pronounced radial density gradient and higher density than wood leading to the lower quantities of oil absorbed by bamboo than wood [17,21]. Therefore, the mechanical properties and mechanisms of oil heat treated wood could not be directly used as a guide for bamboo.

The objective of this study was to determine the effects of tung oil heat treatment on mechanical properties of moso bamboo. For this purpose, moso bamboo samples were heat treated with tung oil at different temperature (100–200 °C) and their mechanical properties were evaluated. Meanwhile, the nanostructures of cell walls of bamboo after heat treatment in tung oil, such as the density, chemical compositions, and cellulose crystalline, were studied in detail to explain the cause of the changes in mechanical behaviors.

## 2. Materials and Methods

### 2.1. Materials

A five years old moso bamboo (Phyllostachys heterocycla) was obtained from Xuancheng, China. Moso bamboo of 1.5 m (height from base) to 3.5 m height was used in this study. Defect-free bamboo materials were dried at room temperature and cut from the center region to 100 mm × 5 mm × 5 mm (longitudinal × tangential × radial). Then, before use, the samples were kept in a climate-controlled room until the moisture content reached approximately 12%. Tung oil was purchased from Emperor’s craftsman, Shanghai, China.

### 2.2. Sample Preparation

Moso bamboo samples were heat treated with tung oil at 100 °C, 140 °C, 180 °C, 200 °C for 3 h, respectively. The modified bamboo was named as T-Oil, for example, 100 °C-Oil is the abbreviation of bamboo after heat treatment in tung oil at 100 °C. During heat treatment of samples, the operating temperature was maintained constant within ± 2 °C. After oil heat treatment, the samples were wiped and then conditioned in a climate-controlled room (at 65 ± 5% relative humidity and temperature of 20 ± 2 °C) to reach equilibrium moisture content. 

### 2.3. Characterization

The morphology of bamboo was characterized by scanning electron microscopy (SEM) (Model XL30, FEI, Hillsboro, OR, USA). Fourier-transform infrared (FTIR) spectra were recorded on a spectrometer (Nicolet iN10, Thermo Scientific, Waltham, MA, USA) over the wavenumber range of 500–4000 cm^−1^ at a resolution of 4 cm^−1^, using the potassium bromide (KBr) pellet method. The crystalline of cellulose was performed by X-ray diffractometer (D8 Advance, Bruker, Billerica, MA, USA). The chemical components of bamboo were analyzed according to the methods of US National Renewable Energy Laboratory (NREL). The acid soluble lignin fraction in the solution was determined by UV−vis spectrometer (752N, Jingke, Shanghai, China), and sugar (glucose, xylose, and arabinose) fractions were analyzed by High-Performance Liquid Chromatography (HPLC) (1200series, Agilent, Santa Clara, CA, USA) and calibrated using standard sugars.

### 2.4. Density

The density (specific gravity) of the samples was measured using the common water displacement method [22,23]. Samples were dried in an oven at 103 ± 2 °C overnight, and the density was measured by electronic balance densitometer (XFMD-12001A, Lichen, Shanghai, China). The density can be calculated by the following Equation (1):

Density = Mass/Volume(1)

### 2.5. Bending Mechanical Characterization

The moso bamboo with a size of 100 × 5 × 5 mm^3^ (longitudinal × tangential × radial) was used to measure the stress and strain using a three-point bending test by an Instron Microtester (5848, Instron, Norwood, MA, USA). Three-point bending set-up was used with a span of 80 mm and a crosshead speed of 4 mm per min. The moisture percentage of the bamboo samples were about 12%. 

## 3. Results

**Figure 1:**
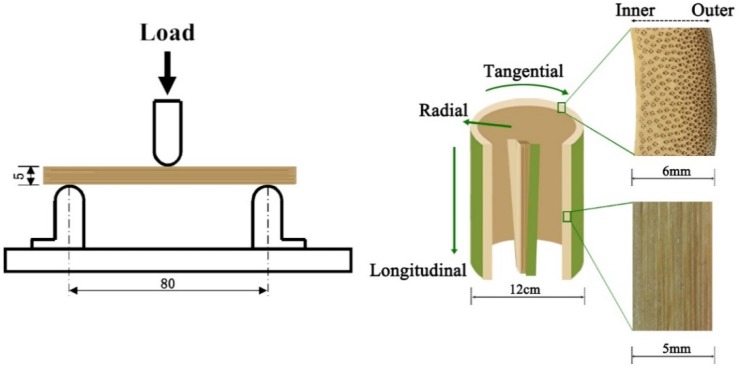
The hierarchical structure of moso bamboo.

Bamboo is a fiber-reinforced porous material, which has naturally evolved to form a hierarchical structure, with fiber density being highest at the outer periphery and lowest at the inner periphery, as shown in Figure 1 [24,25]. Here, bamboo was heat treated with tung oil to improve the less-beneficial features. The mechanical properties of bamboo materials were tested by three-point bending method, and the tangential section flexural behavior was also tested. The flexural behavior in the tangential section would reduce the accidental errors to some extent, which might be caused by sample preparation.

### 3.1. Bending Mechanical Properties

**Figure 2:**
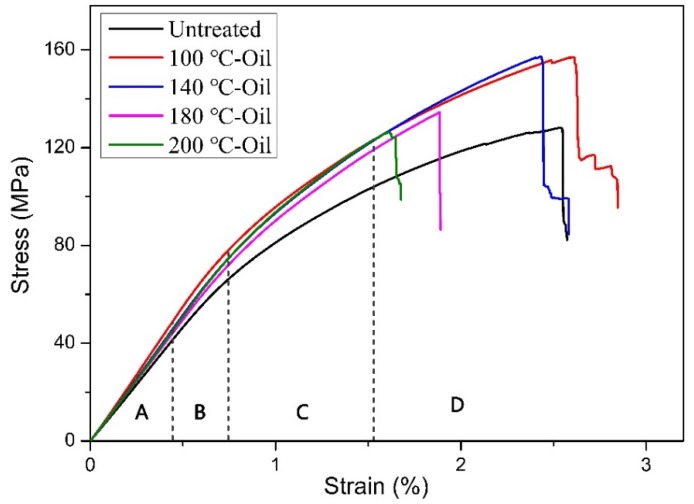
Flexural stress-strain behavior of bamboo composites.

Flexural stress-strain curves of moso bamboo in the tangential section are shown in Figure 2. The stress-strain curve can be divided into four regions. The first region A is known as the linear elastic deformation at low strain; the second region B is related to yielding and nonlinear plastic response; in the third region C, the cell wall of bamboo would collapse in the plastic deformation; and in the region D, as the force was increased, the crack of bamboo propagated continually, finally resulting in serious damage of bamboo. Two mechanical parameters, ultimate strain, and ultimate stress were calculated by the flexural stress-strain curves and the results are listed in Table 1. Ultimate strain implies that the bamboo starts to break at this strain, which results in the failure of bamboo. The ultimate strain slightly increased from 2.55% for untreated bamboo to 2.71% for 100 °C-Oil, but it reduced to 2.43%, 1.83%, and 1.66% for 140 °C-Oil, 180 °C-Oil, and 200 °C-Oil, respectively. The results showed that when bamboo was treated in tung oil at a relatively low temperature (<140 °C), it only had a small impact on the ultimate strain of bamboo material. However, oil heat treatment at a high temperature above 180 °C would lead to an obvious decrease in ultimate strain, probably due to the degradation of hemicellulose, related to the decrease of viscoelasticity [26]. By contrast, the ultimate stress of bamboo was obviously increased after heat treatment in tung oil, compared with the untreated bamboo. The 100 °C-Oil showed the highest ultimate stress, which gradually reduced with further increasing heat treatment temperature up to 200 °C. It is worth to note that the bamboo treated at a high temperature of 200 °C (129.48 MPa) still had slightly higher ultimate stress than that of the untreated bamboo (123.62 MPa).

Table 1 shows that the bamboo after tung oil heat treatment up to 200 °C did not have reduced MOE and MOR, compared to the untreated bamboo. The MOE of 100 °C-Oil was up to 7.6 GPa, obviously higher than the untreated bamboo (6.92 GPa). While the MOE slightly decreased from 7.60 GPa to 7.24 GPa as the heating temperature increased from 100 °C to 180 °C. However, 200 °C-Oil did not further show a reduction in MOE. For MOR, the treated samples also showed generally higher MOR than the untreated sample, which increased from 106.39 MPa for the untreated sample to 149.84 MPa for 100 °C-Oil and then decreased slightly for the samples after heat treatment above 100 °C. The changing trend of MOR was similar to that of ultimate stress. The results indicated that heat treatment in tung oil at a relatively low temperature below 140 °C would not lead to weakening the mechanical performance of bamboo, and most mechanical properties of bamboo were even improved by heat treatment in tung oil at the low temperature. The heat treatment in tung oil at a high temperature over 180 °C presented good mechanical properties of bamboo, particularly in MOE, MOR, and ultimate stress, and showed some decrease in ultimate strain. In order to analyze the reasons for the changes in mechanical behaviors for tung oil heat treated bamboo, the density, chemical compositions, and cellulose crystalline of bamboo were further studied.

### 3.2. Physical Property

**Figure 3:**
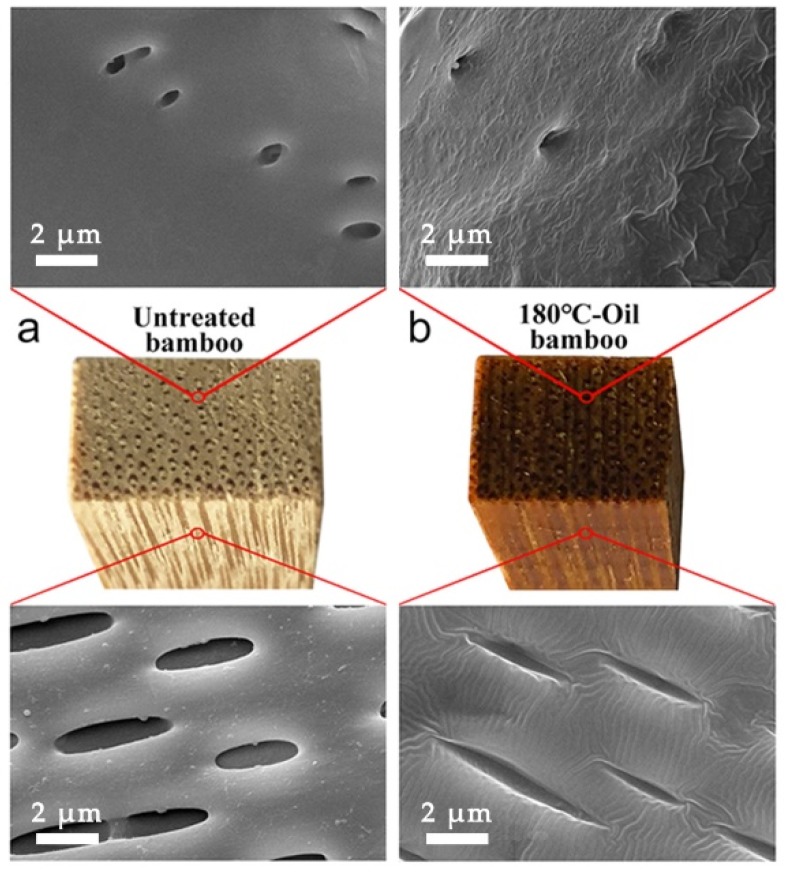
Macroscopic and microscopic morphology of moso bamboo: (**a**) untreated bamboo; (**b**) 180 °C-Oil bamboo.

The density is directly proportional to the mechanical properties of biomass materials [27,28,29], so the density of the untreated and tung oil heat treated bamboo materials were studied and are listed in Table 1. The density of 100 °C-Oil was 0.69 g/cm^3^, higher than that of the untreated bamboo (0.64 g/cm^3^). The results suggested that tung oil had been presented in bamboo after heat treatment. However, the density gradually reduced from 0.69 g/cm^3^ to 0.60 g/cm^3^, with the temperature increasing from 100 °C to 200 °C. The results were mostly in accordance with the changing trends of ultimate stress, MOE, and MOR. Note that the density of 200 °C-Oil (0.60 g/cm^3^) was less than that of the untreated bamboo (0.64 g/cm^3^). The results indicated that the density of bamboo was not only related to tung oil uptake but also to the change in structure and chemical components of bamboo [30,31,32]. Tung oil was obviously observed in the parenchyma cell lumen and trailed in the vessel after tung oil heat treatment (Figure 3), thus further verifying that the tung oil had been presented in bamboo. Mechanical properties of bamboo increased after oil heat treatment at 100 °C, which might be due to tung oil uptake and higher density in some way [21]. The density reduced as the heating temperature increased from 100 °C to 200 °C, which might be one of the reasons for the decrease in mechanical properties.

### 3.3. Chemical Compositions

**Figure 4:**
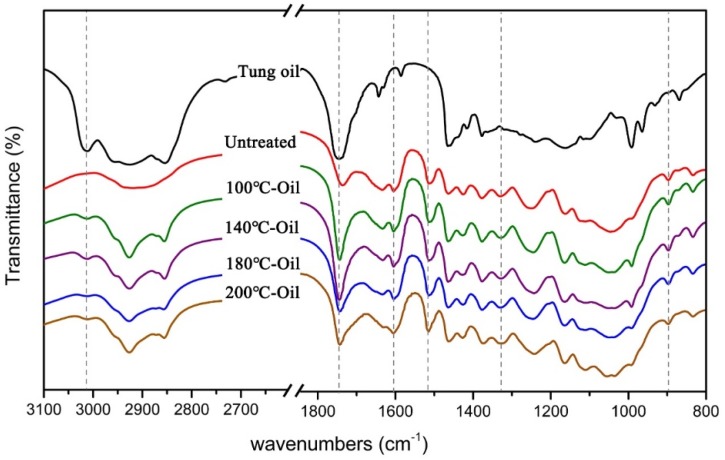
FTIR spectra of moso bamboo.

**Figure 5:**
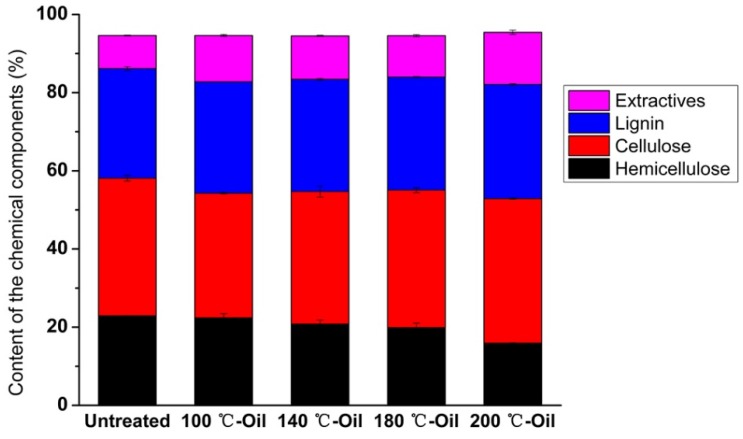
Chemical compositions of untreated and tung oil treated bamboo samples.

The influence of the tung oil heat treatment on the chemical bands of bamboo was investigated by FTIR. As shown in Figure 4, the chemical structure of bamboo remained the same after tung oil heat treatment, while the intensities of many peaks were changed. The absorbance peak at 3010 cm^−1^ in tung oil heat treated bamboo was ascribed to C−H moieties at the carbon-carbon double bond from tung oil [33]. The peak was not found in the untreated bamboo (Figure 4), confirming that the tung oil was presented in modified bamboo. The absorbance peaks at 1510 cm^−1^ and 1600 cm^−1^ in the bamboo were ascribed to aromatic skeletal vibrations from lignin, which showed stronger intensity after heat treatment, particularly, at a heating temperature up to 200 °C, indicating a relative increase of lignin content after oil heat treatment [34]. The peak at 1740 cm^−1^ was assigned to C=O stretching vibration of acetyl groups in hemicellulose or ester groups in tung oil [35], which was slightly reinforced after heating treatment of 100 °C, probably owing to the presence of tung oil in the 100 °C-Oil [36]. Then, the intensity of this band decreased with further increasing temperature, which might be caused by the degradation of hemicellulose. In addition, the peaks at 1326 cm^−1^ and 895 cm^−1^ were almost unchanged, which were assigned to the phenol group and C−H deformation from cellulose, respectively [37]. FTIR results suggested that cellulose content remained stable and hemicellulose content decreased, while lignin content relatively increased after oil heat treatment at a high temperature, which was further supported by chemical composition results (Figure 5).

Bamboo mainly consists of compounds, including cellulose, hemicellulose, and lignin, with different compositions varying by heat treatment [38]. Bamboo mechanical properties probably are influenced by the changes of chemical components [39,40]. Hemicelluloses are a short chain, with amorphous polysaccharides with many acetyl groups, which functioned as the bonding agent [41]. In the process of oil heat treatment, acetic acid was gradually released from acetyl groups, thereby causing acid-catalyzed degradation of amorphous polysaccharides, resulting in hemicellulose reduced from 22.92% for the untreated bamboo to 15.91% for 200 °C-Oil (as shown in Figure 5) [8]. The degradation of hemicellulose would contribute to the significant decrease in ultimate strain at a heating temperature over 180 °C (as shown in Figure 2) [42]. Cellulose is a long-chain polysaccharide with a semi-crystalline structure, which mainly provides the strength to the bamboo. The content of cellulose for the untreated and tung oil heat treated bamboo ranged from 31.88 to 36.99%, and there were no significant differences in the content of cellulose among all bamboo samples (Figure 5). The relatively stable content of cellulose played a positive role in good mechanical performance (e.g. MOE, MOR, and ultimate stress) for the bamboo sample after heat treatment in tung oil at a high temperature. Lignin is an amorphous polymer, whose purpose is to cement the individual cells together and ensures a good capacity of bearing. As lignin is the most heat-resistant component in bamboo, the lignin content slightly increased from 28.49% to 29.22% by gradually increasing the treatment temperature from 100 °C to 200 °C (Figure 5), probably due to condensation and cross-link reactions of lignin or production of compounds featuring aromatic ring products induced by heat treatment. The slight increase in lignin content contributes to the stiffness and strength of bamboo [43]. In summary, the relatively stable content of cellulose and a slight increase in lignin content played a positive role in good mechanical performance. On the other hand, ultimate strain rapidly decreased at a treatment temperature above 180 °C, which might be caused by the obvious degradation of hemicellulose.

### 3.4. Crystallinity of Cellulose

**Figure 6:**
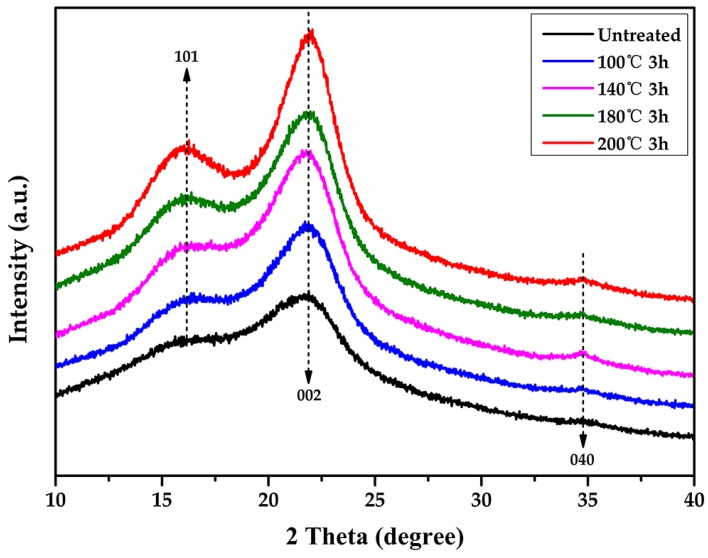
XRD patterns of moso bamboo.

The crystalline region of cellulose is a stable and ordered structure that contributes to its excellent mechanical properties [44]. To further analyze the cellulose structure and the crystallinity index (CrI) of bamboo samples after tung oil heat treatment, XRD patterns were studied, and the results are shown in Figure 6. The untreated and tung oil heat treated bamboo have similar diffraction patterns with three peaks around 16.15° (101), 21.85° (002), and 34.52° (040), which stand for the typical cellulose I structure [45]. This result indicated that the crystal integrity was maintained well after heat treatment in tung oil. However, the diffraction peak of 21.85° became sharper with the increase of treatment temperature, suggesting that bamboo samples became more crystalline. It can be observed that the CrI increased from 33.3% to 44.4% by gradually increasing the temperature from 100 °C to 200 °C, and CrI of the untreated bamboo (24.5%) was obviously lower than that of the oil heat treated sample (Table 1). The increase in crystallinity was probably attributed to the degradation of hemicelluloses (Table 1) and the rearrangement of cellulose, which existed in the amorphous regions [46]. The degradation of the amorphous region would help to improve mechanical performance as the amorphous region is lower in strength than the crystalline region in cellulose [47]. Thus, the increase in bamboo crystallinity after tung oil heat treatment was conducive to excellent mechanical properties [48]. 

Generally speaking, the MOE, MOR, and ultimate stress of moso bamboo after heat treatment with tung oil were higher than the untreated bamboo, but the ultimate strain was significantly decreased at the heating temperature over 180 °C, which might be assigned to the comprehensive effects of the changes in nanostructures of cell walls of bamboo (e.g., density, chemical compositions, and cellulose crystallinity) caused by heat treatment in tung oil. Specifically, heat treatment in tung oil at 100 °C did not cause the degradation of the main chemical compositions of bamboo, but induced a higher density and enhanced CrI, leading to the increase of bending mechanical properties [6,27,49]. When the treatment temperature increased from 100 °C to 200 °C, the mechanical properties of bamboo gradually declined. Although the crystallinity of cellulose was increased by tung oil heat treatment, the degradation of hemicellulose and the decrease of density were responsible for the decrease in mechanical properties. Note that the ultimate strength of bamboo after oil heat treatment was generally higher than that of the untreated bamboo. The main reason is that the cellulose in bamboo provides strength, and the cellulose content remained stable after oil heat treatment. Furthermore, the crystalline of cellulose was increased after oil heat treatment at a high temperature. While the slight decrease of ultimate stress might be due to the decrease of density and partial degradation of hemicellulose, with the heating temperature increasing from 100 °C to 200 °C. The ultimate stress of bamboo depends highly on the content of hemicellulose because the hemicellulose functions as the bonding agent. The MOE of bamboo after heat treatment was generally higher than the untreated bamboo. For example, the MOE of 100 °C-Oil was increased by 9.8% compared to the untreated bamboo. The higher MOE represents a stiffer bamboo. The MOR implies the amount of stress that bamboo can withstand, the variation of MOR is similar to MOE. Besides that, the ultimate strain decreased significantly from 2.71% for 100 °C-Oil to 1.66% for 200 °C-Oil, which was much lower than that of the untreated bamboo (2.55%). This result indicated that bamboo became more brittle after tung oil heat treatment over 180 °C. The dramatic decline in hemicellulose content caused by tung oil heat treatment at a high temperature was mainly due to the decrease in ultimate strain, as it was related to the decrease in viscoelasticity between cells. 

There are some differences in the properties of different biomass materials after oil heat treatment. As reported by Lee et al. [50], when oil palm trunk particleboards were soaked in palm oil for 24 h at room temperature, followed by treatment at 180–220 °C in the air, degradation of hemicellulose and the formation of new lignin network were observed by FTIR. The MOE and MOR of modified oil palm trunk particleboards were decreased by more than 60%, compared with the untreated samples, due to the degradation of urea-formaldehyde (UF) bonding network. Cheng et al. reported [17] that moso bamboo was heat treated with silicon oil at 160, 175, and 190 °C for 2 h. Compared to the untreated bamboo, the MOR of oil heat treated bamboo increased by 5.1% (160 °C and 16.6% (175 °C), mainly due to the oil uptake. But the MOR decreased by 9.6% as the temperature increased to 190 °C. For the sample treated at 190 °C, the degradation of chemical components would play a more important role in the MOR than the limited oil uptake. Mohebby et al. [49] reported that fir wood was heat treated with soybean oil at 100–180 °C for 0.5–1.5 h. The density and MOE of fir wood after oil heat treatment at 180 °C were slightly increased compared to the untreated wood, while the MOR of oil heat treated wood was much higher than the untreated wood. However, few characterizations were investigated to analyze the influence of the nanostructures of cell walls of wood on the mechanical performance after heat treatment in soybean oil. They mainly explain the causes for the changes in mechanical behaviors based on the previous literature, which concluded that cleavage between lignin and hemicelluloses, reorientation, and condensation in the lignin structure along with the formation of new cross-linking might be the main reasons for the increase of MOR. The increase in the MOE might be related to the increase of cellulose crystallinity, because of degradation in amorphous cellulose. In summary, when natural biomaterials were oil heat treated at a low temperature, there was a little change in the chemical compositions, and the mechanical performance increased, mainly due to the oil uptake. While oil heat treatment of biomaterials at a high temperature might result in a decrease in mechanical performance, mainly due to the degradation of chemical components.

## 4. Conclusions

Tung oil heat treatment, as a cost-effective and sustainable modification method, can effectively improve bamboo inherent drawbacks. This work examines the mechanical properties of moso bamboo after tung oil heat treatment, and their influencing factors, such as density, chemical compositions, and cellulose crystalline. The results showed that the 100 °C-Oil had a better mechanical performance than the untreated bamboo, as a result of tung oil uptake, the higher density, and cellulose crystalline, with a little change in the cell structure and chemical components of bamboo. Although the mechanical properties, such as MOE, MOR, and ultimate stress, gradually decreased with treatment temperature rising from 100 °C to 200 °C, they were still higher than the untreated bamboo, probably due to the tung oil uptake, stable cellulose content, and the increment of cellulose crystalline. Besides that, the ultimate strain of bamboo materials did not change remarkably by the thermal treatment in tung oil below 140 °C, but it decreased obviously at the treatment temperature over 180 °C, mainly due to the degradation of hemicellulose resulting in a decrease in the viscoelasticity of the cell wall. Accordingly, bamboo after tung oil heat treatment below 140 °C had a better mechanical performance. Bamboo after tung oil heat treatment over 180 °C was still stiffer and stronger than the untreated bamboo but became more brittle.

## Figures and Tables

**Table 1:** The physical and bending mechanical properties of moso bamboo.

Treatment (°C)	Density (g/cm^3^)	Crystallinity Index (CrI / %)	Bending Mechanical Properties			
			MOE (GPa)	MOR (MPa)	Ultimate Stress (MPa)	Ultimate Strain (%)
Untreated	0.64 (0.02)	24.5	6.92 (0.49)	106.39 (5.82)	123.62 (6.72)	2.55 (0.08)
100	0.69 (0.02)	33.3	7.60 (0.47)	128.14 (4.80)	149.84 (9.08)	2.71 (0.15)
140	0.66 (0.02)	34.0	7.51 (0.54)	128.02 (7.90)	148.69 (10.16)	2.43 (0.12)
180	0.63 (0.03)	35.5	7.24 (0.67)	112.01 (8.68)	130.78 (11.55)	1.83 (0.11)
200	0.60 (0.02)	44.4	7.51 (0.60)	107.88 (8.20)	129.48 (10.70)	1.66 (0.10)

*MOE*: modulus of elasticity; *MOR*: modulus of rupture.
